# The growth and infectivity of *Leishmania major* is not altered by in vitro exposure to 2,3,7,8-tetrachlorodibenzo-*p*-dioxin

**DOI:** 10.1186/s13104-018-3759-x

**Published:** 2018-09-04

**Authors:** Vera Sazhnev, Gregory K. DeKrey

**Affiliations:** School of Biological Sciences, College of Natural and Health Sciences, University of Northern Colorado, 501 20th Street, Greeley, CO 80639 USA

**Keywords:** *Leishmania major*, Dioxin, TCDD, Proliferation, Infectivity

## Abstract

**Objective:**

The numbers of *Leishmania major* parasites in foot lesions of C57Bl/6, BALB/c or SCID mice can be significantly reduced by pre-exposure to 2,3,7,8-tetrachlorodibenzo-*p*-dioxin (TCDD). One potential mechanism to explain this enhanced resistance to infection is that TCDD is directly toxic to *L. major*. This potential mechanism was addressed by exposing *L. major* promastigotes and amastigotes to TCDD in vitro and examining their subsequent proliferation and infectivity.

**Results:**

We found no significant change in the rate of in vitro *L. major* proliferation (promastigotes or amastigotes) after TCDD exposure at concentrations up to 100 nM. Moreover, in vitro TCDD exposure did not significantly alter the ability of *L. major* to infect mice, trigger lesion formation, or survive in those lesions.

## Introduction

Approximately one million new cases of leishmaniasis occur each year worldwide [1, 2]. Of the various forms, cutaneous leishmaniasis is the most common, and visceral leishmaniasis is the most deadly. All forms of the disease are caused by infection with protozoan parasites of the genus *Leishmania*. The most widely studied model of experimental cutaneous leishmaniasis is the *L. major*-infected mouse. Some mouse strains (e.g., C57Bl/6) show resistance to *L. major* infection by killing the parasites and controlling their numbers. Other mouse strains [e.g., BALB/c, and severe combined immunodeficient (SCID)] are lethally susceptible to *L. major* due to insufficient parasite killing, uncontrolled parasite proliferation, and wide-spread dissemination from the site of infection. Killing *L. major* parasites in mice is typically dependent upon Th1 cells supporting NO production by infected macrophages [3–5].

Previous studies in this laboratory have found that the numbers of *L. major* parasites in foot lesions of C57Bl/6, BALB/c or SCID mice can be significantly reduced by exposure to 2,3,7,8-tetrachlorodibenzo-*p*-dioxin (TCDD) prior to infection [6, 7]. TCDD is a coplanar halogenated aromatic hydrocarbon, a prototypical high-affinity agonist of the aryl hydrocarbon receptor (AhR), and the most common AhR ligand used experimentally to investigate the impact of AhR activation on the physiology of animals [8]. No mechanisms have yet been identified to explain the enhanced T cell-independent resistance to *L. major* infection by TCDD in mice. One previously proposed mechanism [6] is that TCDD is directly toxic to *L. major*. We addressed this potential mechanism by exposing *L. major* promastigotes and amastigotes to TCDD in vitro and examining subsequent proliferation and infectivity. We show here that neither proliferation nor infectivity were significantly altered. These results do not support a direct toxic effect of TCDD on *L. major*.

## Main text

### Materials and methods

#### Animals, parasites and TCDD

Female C57BL/6 and BALB/c mice, 6–8 weeks of age, were obtained from breeding colonies at the University of Northern Colorado with breeding stocks originally obtained from Jackson laboratories (Bar Harbor, ME). Animals were maintained on a 12-h light/dark cycle in Optimice^®^ cages containing Tek-Fresh bedding (Envigo, 7099). Animals were provided with food (Envigo, Rodent Diet 2016) and deionized water ad libitum. Euthanasia was performed with an overdose of CO_2_.

*Leishmania major* (LV39, RHO/SU/59/P, Neal, or P strain) promastigotes were maintained by biweekly passage through C57Bl/6 mice followed by re-isolation from foot lesions on a rotator at room temperature in Promastigote Medium consisting of Schneider’s Insect medium supplemented with 10% (v/v) heat-inactivated FBS, 5 µg/mL hemin, 50 µg/mL gentamycin, 100 U/mL penicillin, 100 µg/mL streptomycin, 10 mM Hepes, 116 µg/mL arginine, 36 µg/mL asparagine, 110 µg/mL sodium pyruvate, and 292 µg/mL l glutamine. *L. major* amastigotes were isolated from the feet of C57Bl/6 mice infected with 20 million stationary phase promastigotes 17–21 days earlier using methods described previously [9, 10]. Amastigotes were maintained in Amastigote Medium at 37 °C with humidified air and 5% CO_2_ as described by Wenzel et al. [11].

TCDD was purchased from Cambridge Isotope Laboratories (Andover, MA) and dissolved in dimethyl sulphoxide (DMSO). The concentration of TCDD was confirmed by gas chromatography using the method of Laberton et al. [12].

#### Experimental culture conditions and animal infection

Parasite cultures were initiated in 24-well culture plates with 5 × 10^5^ procyclic promastigotes or 1 × 10^5^ amastigotes per 1.0 mL of medium and maintained for 4 days. Amastigote culture conditions were sufficient to maintain purely amastigote cultures for 2 days after which promastigotes became evident. Control cultures included medium only or DMSO at 0.2% in medium. Treated cultures contained TCDD at concentrations up to 100 nM. This maximum concentration was chosen because it approximates the exposure level that has been shown to reduce *L. major* numbers in wild type mice [6]. Amphotericin B (0.1 mg/mL) served as a positive control for parasite killing. Each culture condition was replicated in triplicate.

To generate stationary-phase promastigotes for infecting mice, procyclic promastigotes were cultured with DMSO or TCDD as described above until stationary phase was achieved. BALB/c mice were then injected with 50 µL of PBS containing one million parasites into one rear foot pad as described previously [7].

#### Data collection and statistics

Parasite cultures were sampled daily. Viable parasites were enumerated by microscopy using trypan blue and a hemacytometer. Mouse foot lesion thickness over time and lesion parasite numbers on day 28 post infection were determined as described previously [6, 13]. Data were analyzed by analysis of variance using SigmaPlot software (version 13) with treatment and experiment number as sources of variation. Values of *P* ≤ 0.05 were considered significantly different.

### Results

#### Promastigote cultures

**Fig. 1:**
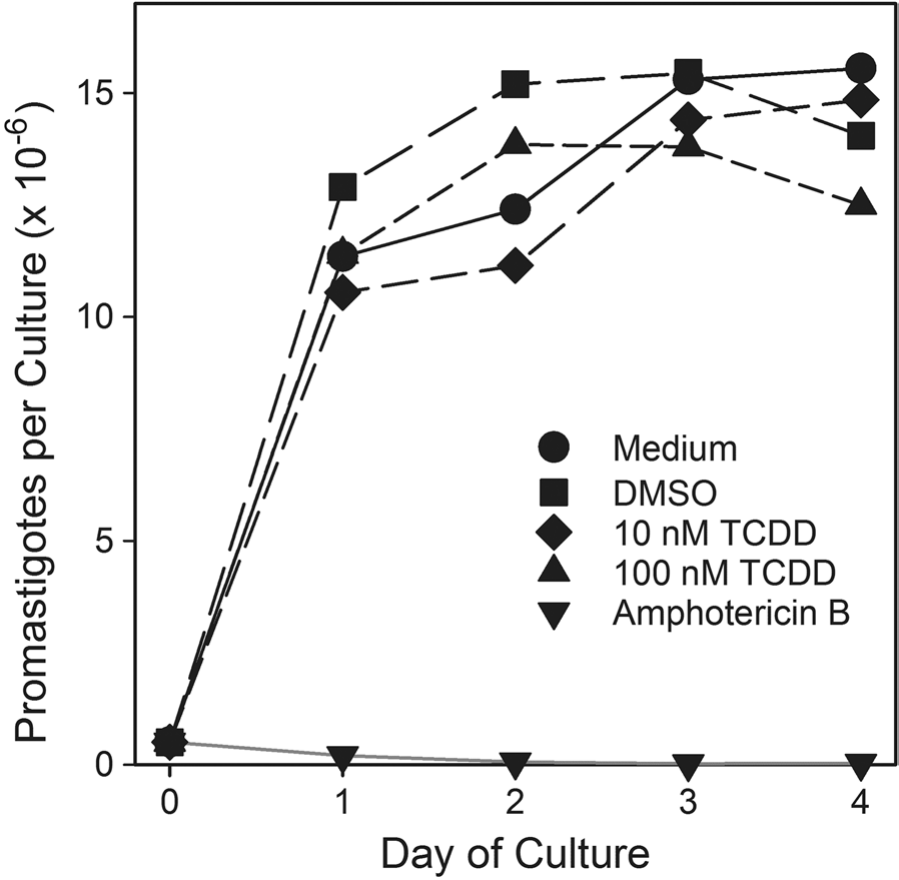
Promastigote in vitro growth curves. Each line represents the average for triplicate cultures under each condition. The data are representative of five independent experiments

The numbers of *L. major* promastigotes per culture in one representative experiment (out of five) are shown in Fig. 1. Both control and TCDD-exposed *L. major* promastigotes proliferated rapidly in experimental cultures reaching stationary phase after approximately 3 days and a maximum concentration of approximately 15 million parasites per mL. In contrast, no increase in promastigote numbers was observed in cultures containing amphotericin B. When the results of five independent experiments were combined and areas under the growth curve (AUC) were calculated for each replicate, no statistically significant differences were found between any treatment groups except in comparison to the amphotericin B treatment (Table 1).

**Table 1:** AUC for promastigote 4-day growth curves

Treatment	Mean*	SEM
Medium only	1^a^	0.62
DMSO	1.09^a^	0.68
10 nM TCDD	0.98^a^	0.62
100 nM TCDD	0.98^a^	0.63
Amphotericin B	− 0.09^b^	0.01

* Mean AUC shown as normalized to medium only control. Means with different letters are significantly different

#### Amastigote cultures

After 2 days of culture, the number of amastigotes in medium-only cultures was 0.63 million ± 0.48 million (mean ± standard error of the mean [SEM]). The number of parasites in the DMSO (0.64 ± 0.29 million) or 100 nM TCDD (0.31 ± 0.18 million) exposed cultures were not significantly different from that found in medium-only cultures or cultures exposed to lower concentrations of TCDD. In contrast, treatment with amphotericin B reduced amastigote numbers by > 95% to 0.027 ± 0.012 million.

#### Mouse foot lesion thickness and parasite numbers

**Fig. 2:**
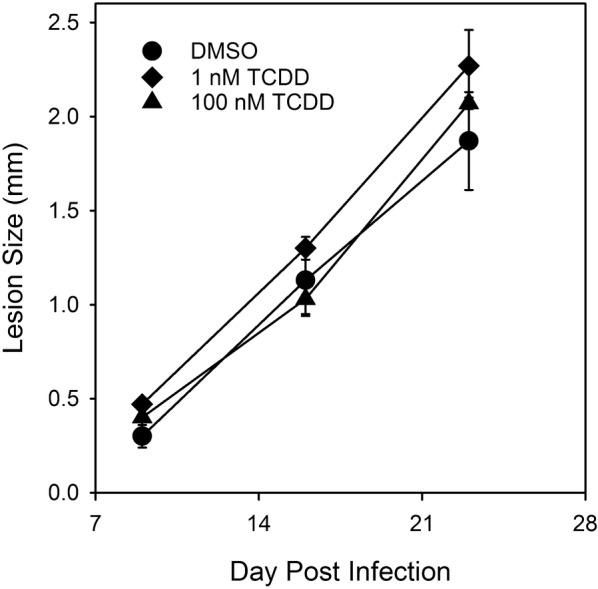
Lesion size in BALB/c mice after infection with treated parasites. Each line represents the average for three mice. The data are representative of two independent experiments

Lesion thickness data from one representative experiment (out of two) are shown in Fig. 2. No significant differences were observed between treatment groups at any time point. The numbers of parasites per foot in control BALB/c mice infected with DMSO-exposed *L. major* 28 days earlier was 15.6 ± 6.4 million per foot (mean ± SEM). The numbers of parasites in the lesions of BALB/c mice infected with *L. major* exposed to 100 nM TCDD was 18.7 ± 3.6 million per foot and was not significantly different from that found in control mice or mice infected with parasites exposed to 1 nM TCDD (16.1 ± 2.1 million).

### Discussion

TCDD exposure is known to cause many changes in cellular function among common vertebrates with most, if not all, of those effects being mediated through TCDD’s action as an agonist of the AhR [8, 14]. Although AhR proteins have been identified in numerous organisms within the kingdom Animalia (but not outside it), the ability to bind planar aromatic hydrocarbons with high affinity appears to be unique to vertebrates [15]. *Leishmania* organisms are eukaryotes, but as trypanosomes they are classified outside the kingdom Animalia, and an AhR has not been reported to exist within them. In this study we demonstrated that TCDD exposure did not significantly impact the rate of *L. major* proliferation in culture as promastigotes or amastigotes. Moreover, TCDD exposure did not alter the ability of *L. major* to infect mice, trigger lesion formation of equivalent size, or survive over 4 weeks to equivalent numbers in those lesions. These results suggest that the reduced numbers of *L. major* that we have observed previously in resistant and susceptible TCDD-treated mice [6, 7] are not caused by direct TCDD toxicity to the parasite.

## Limitations

This study had some limitations. The culture conditions used to maintain *L. major* in vitro were not adequate to prevent amastigote transformation into promastigotes beyond 2 days. Therefore, conclusions as to the sensitivity of *L. major* amastigotes to TCDD are constrained to this time-frame. To our knowledge, no culture conditions have yet been developed to support *L. major* amastigote viability in vitro for extended periods. Because exposures of *L. major* to TCDD were performed in vitro, this study did not model the influence that TCDD may have on *Leishmania* in vivo.

## Abbreviations

AUC: area under the curve
AhR: aryl hydrocarbon receptor
DMSO: dimethyl sulphoxide
SEM: standard error of the mean
TCDD: 2,3,7,8-tetrachlorodibenzo-*p*-dioxin
